# Staging of progressive supranuclear palsy-Richardson syndrome using MRI brain charts for the human lifespan

**DOI:** 10.1093/braincomms/fcae055

**Published:** 2024-02-20

**Authors:** Vincent Planche, Boris Mansencal, Jose V Manjon, Wassilios G Meissner, Thomas Tourdias, Pierrick Coupé

**Affiliations:** 1 Institut des Maladies Neurodégénératives, Univ. Bordeaux, CNRS, UMR 5293, F-33000 Bordeaux, France; Centre Mémoire Ressources Recherches, Service de Neurologie des Maladies Neurodégénératives, Pôle de Neurosciences Cliniques, CHU de Bordeaux, F-33000 Bordeaux, France; CNRS, Univ. Bordeaux, Bordeaux INP, Laboratoire Bordelais de Recherche en Informatique (LABRI), UMR5800, F-33400 Talence, France; Instituto de Aplicaciones de las Tecnologías de la Información y de las Comunicaciones Avanzadas (ITACA), Universitat Politècnica de València, Camino de Vera s/n, 46022, Valencia, Spain; 1 Institut des Maladies Neurodégénératives, Univ. Bordeaux, CNRS, UMR 5293, F-33000 Bordeaux, France; Service de Neurologie des Maladies Neurodégénératives, Réseau NS-Park/FCRIN, CHU Bordeaux, F-33000, Bordeaux, France; Department of Medicine, Christchurch, and New Zealand Brain Research Institute, Christchurch, 8011, New Zealand; Inserm U1215—Neurocentre Magendie, Bordeaux F-33000, France; Service de Neuroimagerie diagnostique et thérapeutique, CHU de Bordeaux, F-33000 Bordeaux, France; CNRS, Univ. Bordeaux, Bordeaux INP, Laboratoire Bordelais de Recherche en Informatique (LABRI), UMR5800, F-33400 Talence, France

**Keywords:** progressive supranuclear palsy, Richardson syndrome, MRI, brain charts, staging

## Abstract

Brain charts for the human lifespan have been recently proposed to build dynamic models of brain anatomy in normal aging and various neurological conditions. They offer new possibilities to quantify neuroanatomical changes from preclinical stages to death, where longitudinal MRI data are not available. In this study, we used brain charts to model the progression of brain atrophy in progressive supranuclear palsy—Richardson syndrome. We combined multiple datasets (*n* = 8170 quality controlled MRI of healthy subjects from 22 cohorts covering the entire lifespan, and *n* = 62 MRI of progressive supranuclear palsy—Richardson syndrome patients from the Four Repeat Tauopathy Neuroimaging Initiative (4RTNI)) to extrapolate lifetime volumetric models of healthy and progressive supranuclear palsy—Richardson syndrome brain structures. We then mapped in time and space the sequential divergence between healthy and progressive supranuclear palsy—Richardson syndrome charts. We found six major consecutive stages of atrophy progression: (i) ventral diencephalon (including subthalamic nuclei, substantia nigra, and red nuclei), (ii) pallidum, (iii) brainstem, striatum and amygdala, (iv) thalamus, (v) frontal lobe, and (vi) occipital lobe. The three structures with the most severe atrophy over time were the thalamus, followed by the pallidum and the brainstem. These results match the neuropathological staging of tauopathy progression in progressive supranuclear palsy—Richardson syndrome, where the pathology is supposed to start in the pallido-nigro-luysian system and spreads rostrally via the striatum and the amygdala to the cerebral cortex, and caudally to the brainstem. This study supports the use of brain charts for the human lifespan to study the progression of neurodegenerative diseases, especially in the absence of specific biomarkers as in PSP.

## Teaser

Planche *et al*. combined multiple MRI datasets to extrapolate lifetime volumetric models for brain structures in healthy aging and progressive supranuclear palsy—Richardson syndrome. They proposed a descriptive MRI staging scheme for progressive supranuclear palsy—Richardson syndrome, comprising six major consecutive stages of atrophy progression that closely align with the neuropathological staging of tauopathy progression.

## Graphical Abstract

**Graphical abstract:**
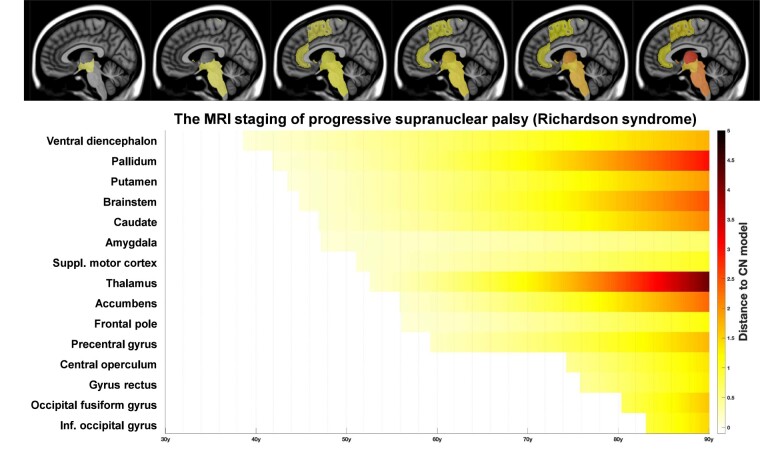

## Introduction

Progressive supranuclear palsy (PSP) is a neurodegenerative disease with a heterogeneous clinical presentation. The neuropathological diagnosis is based on the presence of neuronal and glial 4R-tau inclusions. Richardson’s syndrome (PSP-RS), with key features of supranuclear gaze palsy and postural instability, is the classical PSP phenotype and remains the most frequent syndromic presentation according to recent clinico-neuropathological studies.^1^ The clinical progression of PSP is associated with the spreading of tau pathology along connected neuronal pathways, as suggested by correlative studies combining functional MRI, tau-PET and post-mortem neuropathological assessment in patients with PSP,^2^ and also by more direct experimental evidence in macaques injected with patient-derived tau aggregates.^3^

In PSP-RS, a neuropathological staging system of disease progression has been recently proposed. It defines six sequential stages, where tau pathology starts in the pallido-nigro-luysian system and spreads rostrally via the striatum and the amygdala to the cerebral cortex, and caudally to the brainstem and the cerebellum.^4^ This staging system, which has been replicated and validated,^5^ was built on post-mortem neuropathological examination of deceased PSP donors, precluding reliable conclusions about the early stages of disease progression. Providing insights into the early temporo-spatial spreading of PSP pathology is crucial for early diagnosis and the development of disease-modifying therapies.

The use of tau-PET might theoretically help us in the future to describe the anatomical progression of PSP *in vivo.*^6^ That being said, current tau tracers, including second-generation ligands such as [^18^F]PI-2620 exhibit an heterogeneous binding and have limited sensitivity for reliable detection of 4R tauopathy, in contrast to Alzheimer’s disease tauopathy.^7,8,9^ Moreover, longitudinal PET studies in PSP are scarce.^10^ Since brain atrophy correlates with the progression of tauopathy, structural MRI remains a reliable and affordable marker of PSP progression that outperforms first-generation tau-PET.^10^ Longitudinal MRI studies have highlighted the progression of atrophy in PSP-RS, mainly in the midbrain and the frontal lobe.^10,11,12^ These studies cover disease progression over a span of 6–24 months. Unfortunately, achieving a longer follow-up is challenging in the field of PSP, primarily due to factors such as late diagnosis and attrition. Consequently, it is essential to consider alternative methodological approaches to comprehensively depict the entire course of PSP-RS, from its prodromal stages to death.

A probabilistic event-based model was recently applied to cross-sectional MRI to identify the most probable sequence of regional atrophy over the course of PSP-RS.^13^ This *in vivo* ordering of structural progression of PSP-RS corresponds broadly to the post-mortem neuropathological staging of tauopathy but with some notable exceptions, such as late atrophy of the pallidum, a structure among the first affected by the underlying tauopathy.^14^ As a main limitation, these cross-sectional event-based models have no explicit timescale, and are unable to describe the time to transition between each anatomical stage. While models based on temporal events have been recently proposed to infer timelines of pathological events,^15^ they require longitudinal MRI, which is a scarce resource; therefore, they have never been used to study PSP. Furthermore, these event-based models cannot infer the prodromal/preclinical stages of the disease (i.e. before the occurrence of the first measurable event in patients).

In this study, we proposed a different modelling approach to define the structural progression of PSP-RS over the entire course of the disease. Indeed, recent advances in BigData sharing in neuroimaging have enabled the emergence of brain charts for the human lifespan,^16,17^ where large numbers of cross-sectional MRI are used to build extrapolated dynamic models of brain anatomy. Such standards for human brain measurement offer new possibilities to quantify neuroanatomical changes and to map the transition from normal aging to early stages of neurodegenerative diseases. Thanks to this methodology and its validation using ‘truly’ longitudinal data, we have recently proposed the anatomical MRI staging of Alzheimer’s disease^18^ and the three clinical variants of frontotemporal dementia (FTD).^19^ Here, we describe the progressive pattern of neuroanatomical variations between PSP-RS and normal aging.

## Methods

### Standard protocol approvals, registrations and patient consents

All data were obtained in de-identified format upon request from external study centres, who ensured compliance with ethical guidelines. All subjects included in the MRI databases used in this study provided informed consent. The protocol for each study/cohort was approved by the institution review board at all sites (see the Acknowledgments section).

### Datasets

Healthy and PSP-RS trajectories of brain atrophy were estimated thanks to the aggregation of 22 open-access datasets. We collected a total of 8318 T1-weighted MRIs scanned on 1.5T or 3T magnets.

After quality control (see below), 8170 MRIs from healthy subjects, covering the entire lifespan (from 1 to 100 years of age) were included in the study. The 22 cohorts with healthy subjects used in this study are listed in Table 1. References and websites are listed in the Acknowledgements section.

**Table 1:** Complete list of the cohorts used in this study

DATASET	Group	*n*	Gender	Age range (years)
C-MIND (Cincinnati MR Imaging of Neurodevelopment)	Controls	236	F = 129/M = 107	0.7–19
NDAR (National Database for Autism Research)	Controls	382	F = 174/M = 208	1–50
ABIDE (Autism Brain Imaging Data Exchange)	Controls	492	F = 84/M = 408	6–52
ICBM (International Consortium for Brain Mapping)	Controls	294	F = 142/M = 152	18–80
IXI (Information eXtraction from Images)	Controls	549	F = 307/M = 242	20–86
ADNI 1&2 (Alzheimer’s Disease Neuroimaging Initiative)	Controls	404	F = 203/M = 201	60–90
AIBL (Australian Imaging Biomarkers and Lifestyle Study of Ageing)	Controls	232	F = 175/M = 157	55–93
ADHD-200 (Attention-Deficit Hyperactivity Disorder-200 Consortium)	Controls	544	F = 263/M = 281	7–26
DLBS (Dallas Lifespan Brain Study)	Controls	315	F = 198/M = 117	21–89
ISYB (Imaging Chinese Young Brains)	Controls	213	F = 155/M = 58	18–30
MIRIAD (Minimal Interval Resonance Imaging in Alzheimer’s Disease)	Controls	23	F = 11/M = 12	58–86
PPMI (Parkinson’s Progression Markers Initiative)	Controls	166	F = 61/M = 105	31–83
PREVENT-AD (Pre-symptomatic Evaluation of Experimental or Novel Treatments for Alzheimer’s Disease)	Controls	307	F = 215/M = 92	55–84
AOMIC (Amsterdam open MRI collection)	Controls	1361	F = 731/M = 630	18–26
Calgary preschool MRI dataset	Controls	263	F = 115/M = 148	3–7
CamCAN (Cambridge Centre for Ageing and Neuroscience)	Controls	653	F = 330/M = 323	18–89
PIXAR	Controls	155	F = 84/M = 71	4–39
SALD (Southwest University Adult Lifespan *Dataset)*	Controls	494	F = 307/M = 185	19–80
SRPBS (Japanese Strategic Research Program for the Promotion of Brain Science)	Controls	791	F = 365/M = 426	18–80
NACC (National Alzheimer’s Coordinating Canter)	Controls	161	F = 112/M = 49	30–100
NIFD (NeuroImaging in Frontotemporal dementia)	Controls	135	F = 76/M = 59	39–81
4RTNI (4-repeat tauopathy neuroimaging initiative)	PSP-RS	62	F = 36/M = 26	55–86

We combined the control groups of 22 cohorts covering the entire lifespan to model healthy trajectories and patients from the 4RTNI cohort to extrapolate lifetime volumetric models of PSP-RS.

For PSP-RS, we used MRI from the 4R Tauopathy Imaging Initiative (4RTNI, https://4rtni-ftldni.ini.usc.edu/). Patients with PSP in this dataset met the National Institute of Neurological Disorders and Stroke (NINDS) for PSP-RS criteria.^20^ We included 62 patients with PSP in this study and all were retained after quality control (Tables 1 and 2).

**Table 2:** Final datasets description

	Healthy controls	PSP-RS
Number of subjects	8170	62
Age (years), mean [range]	36.8 [0.7–100]	70.4 [55–86]
Sex	F = 4160; M = 3997	F = 36; M = 26
Disease duration (years), mean [range]	–	5.5 [1–17]
Total PSP rating scale, mean [range]	–	37.9 [10–86]
MoCA, mean [range]	–	20.3 [1–28]
MMSE, mean [range]	–	24.8 [1–30]

This table provides the total number (*n*) of considered images (after quality control), the average ages of participants and intervals in brackets for the gender proportion. It also provides the clinical characteristics of patients with PSP-RS included in the 4RTNI cohort. MoCA, Montreal Clinical Assessment; MMSE, Mini Mental State Examination; PSP, Progressive Supranuclear Palsy; PSP-RS, PSP-Richardson Syndrome.

### Image processing

All the T1-weighted MRI were processed with AssemblyNet (freely available at https://github.com/volBrain/AssemblyNet/).^21^ This software produces whole-brain segmentation of fine-grained structures using a large ensemble of deep neural networks. AssemblyNet is robust to acquisition protocols, age of subjects and presence of brain pathology.^21^ All images were preprocessed to locate them into a common geometrical and intensity space. The preprocessing steps started with denoising,^22^ then the images were corrected for inhomogeneity^23^ and affine-registered into the Montreal Neurological Institute (MNI) space using ANTS.^24^ Finally, a tissue-based intensity normalization was used.^25^ For the segmentation process, the intracranial cavity was segmented using DeepICE method.^26^ Afterwards, structure segmentation was achieved using 250 U-Nets through a multi-scale framework.^21^

All images were automatically quality controlled using the artificial intelligence-based method RegQCNET.^27^ After this first check, a human-based multi-stage quality control procedure was performed blinded to the subject’s group, as previously described.^16,28^ A visual assessment was done for all input images by checking screen shots of one sagittal, coronal and axial slices in the middle of the 3D volume. Images were rejected if partial head coverage, motion artefact, high distortion or abnormal noise level was detected. Then, a visual assessment of processing quality was carried out using the segmentation report, which provides screenshots for each pipeline step. Images were rejected after this step in case of inaccurate registration in the MNI space, inaccurate intracranial cavity extraction, missing brain structures or over/under-segmentation of brain structures. A last control was performed by individually checking all outliers (values higher/lower than 2 SD of the estimated model). For each outlier, the segmentation map was re-inspected using a 3D viewer (ITK-SNAP). In case of segmentation failure, the subject was removed from the study.

On the structures produced by AssemblyNet following the Neuromorphometrics labels,^29^ we considered the 60 left and right grey matter regions: 9 subcortical structures, 17 frontal gyri/lobules, 8 temporal gyri/lobules, 6 parietal gyri/lobules, 8 occipital gyri/lobules, 6 gyri in the limbic cortex, 5 sub-regions of the insular cortex and cerebellar grey matter. We also analysed 4 central structures: the brainstem and three groups of vermal structures (i.e. vermis I-V, vermis VI-VII and vermis VIII-X). As our goal was to describe the anatomical progression of atrophy in the brains of patients with PSP, testing the parallel with the presumed progression of tauopathy, we did not include ventricular volume in our analyses, even though it was provided by AssemblyNet. Because our preliminary analyses did not show evident asymmetry, left and right volumes of symmetric brain structures were added to obtain a global volume.

### Modelling brain charts and statistical analyses

To limit for the variability introduced by head size difference, models were estimated on normalized volumes (% of total intracranial volume). Moreover, we used z-scores of normalized volumes to compare structures of different sizes. The normal distribution of each normalized volume was tested using Kolmogorov–Smirnov test at 95%. Statistics were performed with Matlab using default parameters.

To study brain volumetric trajectories of PSP-RS across the entire lifespan and to extrapolate the early stages of PSP-RS, we followed the strategy we have previously proposed for Alzheimer’s disease and FTD.^18,19^ Our framework was based on the assumption that neurodegeneration is a continuous and progressive process along disease progression. Therefore, to constrain the volumetric trajectories over the entire lifespan, we built our PSP-RS models using control MRIs taken before the age of the youngest patient. Different strategies were then considered to model the healthy and pathological trajectories of each brain structure over time, as previously described.^28^ Briefly, the candidate models were tested from the simplest to the most complex: (i) a linear model; (ii) a quadratic model; and (iii) a cubic model. A model was kept as a potential candidate only when simultaneously F-statistic based on ANOVA (i.e. model versus constant model) was significant (*P* < 0.05) and when all its coefficients were significant using t-statistic (*P* < 0.05). We finally used the Bayesian Information Criterion (BIC) to compare the candidate models and we selected the model providing the lowest BIC. This model selection procedure was applied to all the considered structures.

**Figure 1:**
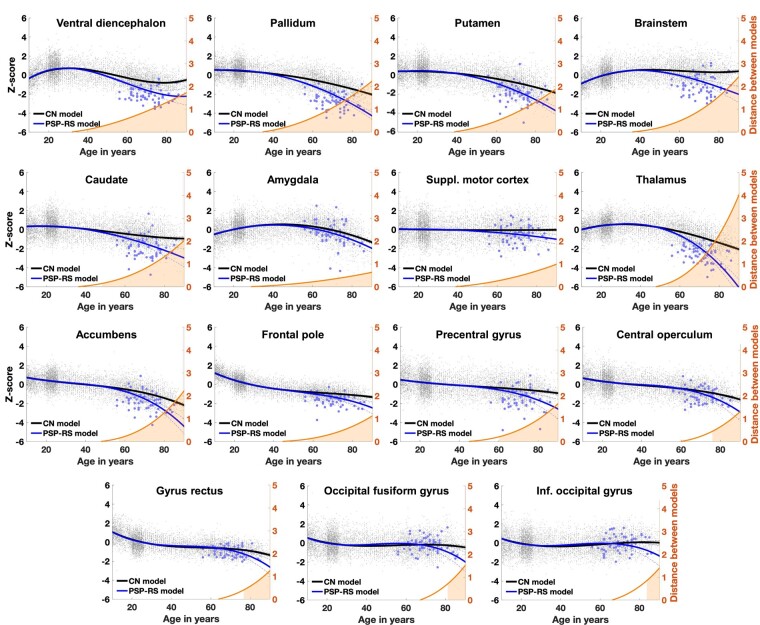
**Lifespan trajectories based on z-scores of normalized brain volumes for cognitively normal (CN) subjects (in black) and patients with PSP-RS (in blue).** Black dots represent all healthy individuals and blue dots patients with PSP-RS. The orange curves represent the distance between the healthy and PSP-RS models. The orange areas indicate the time period where confidence intervals at 95% of both models do not overlap. Only models detected as significantly different between healthy aging and PSP-RS are presented in this figure. In AssemblyNet, the ventral diencephalon regroups the hypothalamus, the mammillary bodies, the subthalamic nuclei, the substantia nigra, the red nuclei and the geniculate nuclei. Suppl: Supplementary; Inf: Inferior.

**Figure 2:**
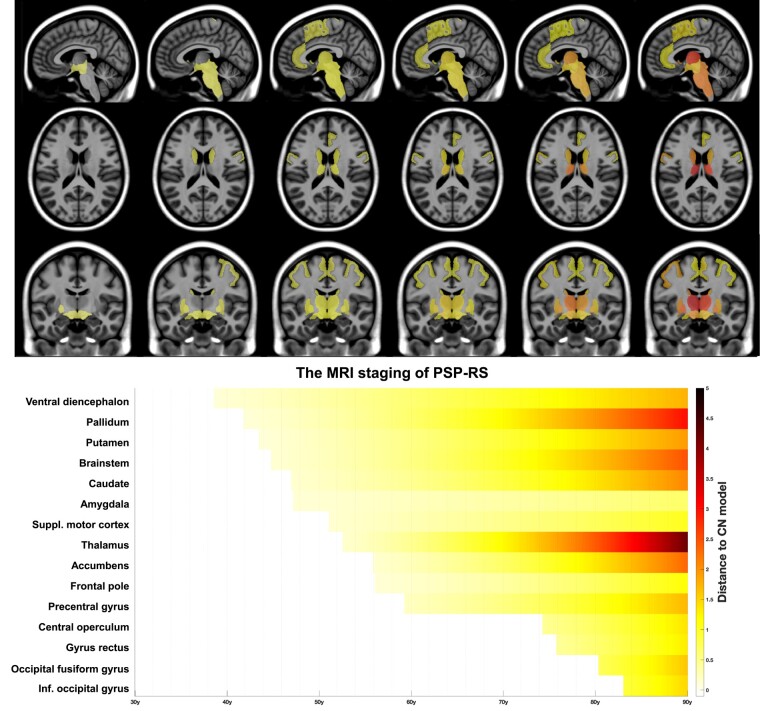
**The MRI staging scheme of PSP-RS.** The upper panel maps the progression of atrophy in the three axes (all brain structures identified in Fig. 1). The lower panel is a timeline representing the sequential divergence of significantly atrophied structures between healthy and PSP-RS volumetric trajectories. The effect-size of structural divergence is color-coded according to the bar at the bottom right of the figure. In AssemblyNet, the ventral diencephalon regroups the hypothalamus, the mammillary bodies, the subthalamic nuclei, the substantia nigra, the red nuclei and the geniculate nuclei. Suppl: Supplementary; Inf: Inferior; CN: cognitively normal.

Afterwards, distances between healthy and PSP-RS trajectories were computed on the estimated models. The prediction bounds were estimated with a confidence level of 95%. A brain structure was considered to be significantly smaller in PSP-RS compared to healthy aging when the two structural trajectories diverged and when their 95% confidence intervals no longer overlapped (Fig. 1). This approach is a conservative version of the *t*-test that compensates for multiple comparisons since the *t*-test can be significant when 95% confidence intervals overlap, while it is always significant when 95% confidence intervals do not overlap. Then, all divergent structures were mapped across time and space on standardized sagittal, coronal and axial MRI planes (Fig. 2). Finally, the sequence of significant divergence of the affected brain structures was listed in chronological order to obtain the MRI staging scheme of PSP-RS (Fig. 2).

## Results

### Dataset description

To study the brain volumetric trajectories of healthy controls and PSP-RS across the entire lifespan, we compiled several open-access databases to construct two datasets. Their composition and characteristics are described in Table 2. For PSP-RS, we used MRI from the 4RTNI. The patients with PSP-RS represented various disease duration and a large spectrum of disease severity, as assessed by the total score of the PSP rating scale^30^ ranging from 10/100 to 86/100. Regarding cognitive impairment, the mean MoCA was 20.3 and the mean Mini Mental State Examination (MMSE) was 24.8, with also a large range of disease severity (Table 2).

After quality control, 8170 MRI from healthy controls remained for the analyses and 62 MRI from patients with PSP-RS. We built our lifespan PSP-RS models by combining MRI of patients with PSP-RS with MRI of healthy controls because we assumed that neurodegeneration is a continuous and progressive process along the pathology evaluation. Herein, we combined the MRIs of PSP-RS patients with MRIs of 5743 healthy controls younger than 55 years (the age of the youngest PSP patient) to build our lifespan PSP-RS model. Consequently, the parametric PSP model was constrained over the entire lifespan using 5805 subjects.^28^

### Identification of brain structures diverging between healthy subjects and PSP-RS trajectories

We identified 15 brain structures (over the 64 grey matter structures tested using our segmentation pipeline) that significantly diverged during lifespan between PSP-RS and healthy aging models (Fig. 1). The three most affected structures over time were the thalamus (distance between the healthy aging and our PSP-RS model at 90 years = 4.0), followed by the pallidum (distance = 2.9), and the brainstem (distance = 2.4).

### The MRI staging scheme of PSP-RS

We mapped in time and space on standardized brains the diverging brain structures between PSP-RS and normal brain charts (Fig. 2, upper panel). We also built a timeline highlighting the sequential progression of brain atrophy in PSP-RS (Fig. 2, lower panel). Schematically, six major stages of atrophy progression can be observed on this timeline, based on the order of divergence, the proximity of structures, as well as the grouping of anatomical substructures (e.g. putamen + caudate + accumbens = striatum; supplementary motor cortex + frontal pole + precentral gyrus + operculum + gyrus rectus = frontal cortex): (i) the ventral diencephalon (regrouping the hypothalamus, the mammillary bodies, the subthalamic nuclei, the substantia nigra, the red nuclei and the geniculate nuclei); (ii) the pallidum; (iii) the brainstem, the striatum and the amygdala; (iv) the thalamus; (v) the frontal lobe; and (vi) the occipital lobe.

## Discussion

In this study, we combined multiple large-scale MRI datasets and whole-brain segmentation of fine-grained structures using a large ensemble of deep neural networks to describe the chronological structural progression of PSP-RS over decades. We found six major consecutive stages of atrophy progression: (i) the ventral diencephalon (including the subthalamic nuclei, the substantia nigra, and the red nuclei); (ii) the pallidum; (iii) the brainstem, the striatum and the amygdala; (iv) the thalamus; (v) the frontal lobe; and (vi) the occipital lobe. The most severely affected structures during the entire course of PSP-RS were the thalamus, followed by the pallidum and the brainstem.

This MRI staging scheme of atrophy progression is very close to the sequence of tau pathology in PSP-RS.^4^ More specifically, our *in vivo* staging of atrophy progression overlaps with the sequence of neuronal tau pathology described in post-mortem neuropathological studies, which begins in the globus pallidus, the subthalamic nucleus, and the substantia nigra (step 1), then accumulates in the midbrain and the pons (step 2), the striatum and the amygdala (step 3), the frontal lobe (step 4), the parietal and temporal lobes (step 5) and finally the occipital cortex (step 6). These results further support the association between brain atrophy and the progression of neuronal tau pathology in PSP, as in other primary or secondary tauopathies.^31,32^ However, the presence of the thalamus in our staging scheme (stage 4), and the severity of its atrophy over the entire course of the disease, suggests that glial pathology also plays a role in the progression of atrophy in PSP-RS. Indeed, astroglial and oligodendroglial tau pathology is described in the thalamic nuclei from step 3 in the neuropathologal staging scheme.^4^ These findings are consistent with studies comparing *pre-mortem* MRI volumetry to post-mortem neuropathology, which reported a correlation between glial tau lesions and focal atrophy in PSP.^33^ The severity of thalamic atrophy is well supported by previous SPECT studies measuring the vesicular acetylcholine transporter expression and showing alterations of the pontothalamic cholinergic pathways that increased with disease progression at both cell body and terminal levels.^34^ The severe but relatively late atrophy of the thalamus also suggests secondary neurodegeneration due to disconnection.

While waiting for new generation tau-PET tracers with good sensibility and specificity for 4R tauopathies, brain charts for the human lifespan appear as a relevant strategy to assess the sequential progression of PSP-RS. Probabilistic event-based modelling is an alternative method to infer the order in which biomarkers become abnormal using cross-sectional data. These data-driven models may be used to estimate the sequence in which brain atrophy progresses using structural MRI and have been applied to many neurodegenerative diseases^35^ including PSP-RS.^13^ These probabilistic models are interesting for automatically detecting biomarker abnormalities and estimating their sequence of occurrence. However, they are not designed to study preclinical/prodromal stages when dedicated biomarkers for these stages (or genetic determinants) are not available. Furthermore, event-based models are ordinal and are not able to draw smooth evolution of volumes according to disease duration.

Importantly, our MRI staging based on PSP-RS brain charts mirrored more closely to the neuropathological staging of the disease than event-based models. This is especially the case regarding the detection of early pallidal atrophy.^14^ Contrary to our model and the neuropathological staging, the event-based models indeed report late pallidal atrophy and place brainstem atrophy first, which we find to occur later, in agreement with the progression of the tauopathy in neuropathologically confirmed cases.^13^ Subtype and Stage Inference (SuStaIn) is another framework that could theoretically allow the study of the temporal progression of brain atrophy with cross-sectional data.^36^ This method simultaneously infers patient sub-groups and the corresponding trajectories of disease progression. So far, this unsupervised machine-learning technique has mainly used to cluster the heterogeneity of atrophy progression in PSP^37,38^ and other tauopathies.^36^

Compared to previous event-based modelling of PSP-RS, another advantage of our MRI staging approach is the establishment of a timescale of atrophy progression. This provides a more precise indication of the time for the disease to progress, including in its preclinical or prodromal phases. For instance, we found that the early nigro-luysian atrophy precedes brainstem atrophy by 6 years in PSP-RS (Fig. 2). This timeframe echoes our previous findings using the same methodological approach in the three FTD variants where subcortical atrophy preceded focal atrophy in specific behavioral and/or language networks by 8–10 years.^19^ Although we do not have MRI data for PSP-RS patients under 55 years old, the significant divergence of our PSP-RS model from the healthy subjects model is reported around 40 years for the ventral diencephalon. Interestingly, this age corresponds to the minimum age required by the Movement Disorder Society in the diagnostic criteria for PSP, in relation to the earliest cases with histological confirmation.^1^ These results are important in terms of internal validity regarding our statistical inference of the earliest stages of the disease.

Beyond these anatomical descriptions, it will be interesting to develop in the future a novel framework for automatic PSP-RS detection using normative and pathological lifespan models, as we previously proposed for Alzheimer’s disease with the hippocampal-amygdalo-ventricular atrophy score.^39^ A future multi-pathology algorithm will be able to leverage the brain charts that we have developed for several neurodegenerative diseases now.^18,19^ It will then be important to assess if the use of these brain charts will allow to differentiate PSP from other atypical parkinsonism and/or from other disorders of the FTLD spectrum.

It would have been interesting to compare the progression trajectories of other phenotypic presentations of PSP, as we have previously reported for the three FTD syndromic variants.^19^ However, existing MRI databases essentially contain data from patients diagnosed with the NINDS-SPSP criteria, *i.e.* with Richardson Syndrome. It is important to point out that this clinical presentation is highly specific to PSP neuropathology, as validated by autopsy, unlike other PSP phenotypes.^1^ In the absence of relevant 4R-tau biomarkers, this allows us to assume high diagnostic accuracy in our sample without post-mortem confirmation to propose the MRI staging scheme of PSP-RS, which is a strength of the present work.

An important limitation of this study is the lack of a fine-grained assessment of cerebellar anatomy. Indeed, the dentate nucleus is affected by tau pathology during the caudal spreading of the disease.^4^ Furthermore, atrophy of the superior cerebellar peduncles (closely related to the dentate nucleus) has long been reported in PSP^40^ and has been proposed as a biomarker to distinguish PSP from other Parkinsonian disorders.^41^ In the present study using AssemblyNet following the Neuromorphometrics labels, we only segmented the whole cerebellar grey matter and three groups of vermal structures (i.e. vermis I-V, vermis VI-VII and vermis VIII-X) but not the cerebellar peduncles. Although our analyses regarding these anatomical structures showed a trend for atrophy in our lifespan models, the divergence between healthy and PSP trajectories was not statistically significant (data not shown). Future studies using brain charts will need to address this anatomical question better.

Another potential limitation of this study concerns the modelling of atrophy progression in relation to patients’ age rather than the duration of the disease or the severity of the disease. Our primary assumption is indeed that a younger patient may have less atrophy than an older patient. This approach is also a strength of the study because it enables us to extrapolate the preclinical/prodromal stages of the disease, which other methods based on cross-sectional data from symptomatic patients do not allow. This assumption could be questioned in Alzheimer’s disease, where the atrophy pattern differs between early-onset and late-onset Alzheimer’s disease.^42^ However, these differences are also explained by different syndromic presentations between early-onset and late-onset Alzheimer’s disease, which is different here in a very specific population of patients with PSP presenting a Richardson phenotype. Furthermore, we have previously demonstrated that this modelling strategy corresponds well to the atrophy progression as described by ‘truly’ longitudinal data.^18^ Finally, if we consider the example of FTD variants, our results regarding the modelling of the earliest stages of atrophy progression align well with what is reported in the literature for pre-symptomatic individuals carrying causal mutations.^19^ Taken together, all these elements make us fairly confident about the extrapolation of the MRI staging we are proposing for PSP-RS, which is supported by neuropathology.^4^ We hope that this MRI staging scheme will help better characterize PSP-RS, amidst clinical criteria and, hopefully, future *in vivo* biological markers.

To conclude, we have modelled the global structural progression of PSP-RS over the entire course of the disease. We proposed a descriptive MRI staging scheme that matches the neuropathological staging of tauopathy progression in PSP-RS, where the pathology starts in the pallido-nigro-luysian system and spreads rostrally via the striatum and the amygdala to the cerebral cortex, and caudally to the brainstem. This study further supports the use of structural MRI and brain charts for the human lifespan to study the progression of neurodegenerative diseases, especially in the absence of specific biomarkers as in PSP.

## Data Availability

MRI raw data from the different cohorts are available online (see Acknowledgments). Other data are available from the corresponding author upon reasonable request.
